# Supplementary material for: Necessity of strengthening the current clinical regulatory for companion diagnostics: An institutional comparison of the FDA, EMA, and MFDS
Source: Mol Ther Methods Clin Dev. 2023 Aug 16;30:447–58. doi: 10.1016/j.omtm.2023.08.008 (PMC10474566; doi:10.1016/j.omtm.2023.08.008)
Supplement: Document S1. Figures S1 and S2 and Tables S3–S8 [file mmc1.pdf]

OMTM, Volume *30*

Supplemental information

Necessity of strengthening the current clinical regulatory for companion diagnostics: An institutional comparison of the FDA, EMA, and MFDS

Su Lim Kang, Jae Hyun Woo, Na Hyeon Kim, Ji Yean Kwon, and Sung Min Kim

Supplemental Material

Table S1. Approved CDx list in FDA

Table S2. Approved CDx list in MFDS

Table S3. Factors that may affect clinical investigation design

[21 CFR 860.7] Determination of safety and effectiveness	
**Purpose**	- A clear statement of the purpose of the study
**population selection**	- Confirmation of the suitability of the subject for the purpose of the study, criteria for the condition to be treated or diagnosed, laboratory tests, if applicable, and, in the case of a device intended to prevent a disease or condition, evidence of susceptibility to and exposure to that condition<br>- Allocate subjects in a way that minimizes possible bias<br>- Demonstration of comparability between the test group and all control groups of relevant variables such as gender, severity or duration of disease, and non-instrumental method
**Parameter**	- Description of methods for observing and recording outcomes, including variables measured, quantification, assessment of subjects' responses, and procedures chosen to minimize subject and observer bias
**Blinding**	- Comparison of treatment or diagnostic results with a control group in a manner that allows for quantitative evaluation<br>- A description of the methods specified and used to minimize the bias of observers and data analysts<br>- Document the level and method of blinding<br>: No treatment. Comparison of objective outcomes in comparable treatment and noncomparative patient groups, provided that objective measures of efficacy are available and the placebo effect is negligible<br>: Placebo control. Where there may be a placebo effect in device use, comparison with an ineffective device used under designed conditions that are as close as possible to the conditions of use under investigation<br>: Active treatment control. Comparison when an effective regimen can be used for comparison<br>: Historical control. Quantitative comparison with past prior experience in the case of specific circumstances associated with high mortality and predictable disease or duration, signs and symptoms of severity, and predictable prophylaxis
**Analysis of results**	- Summary of methods of analysis and evaluation of data derived from the study, including appropriate statistical methods used

Table S4. Diagnostic clinical performance study

	Diagnostic clinical performance study
**Intended Use**	- The study design of the diagnostic clinical performance study depends on the intended use of the device.<br>- Therefore, it should be evaluated in the following items corresponding to the purpose of use.<br>• What the device measures or detects<br>• What the device reports<br>• The cell, tissue, organ, part or system examined<br>• Sample Source, Sample Type, and Sample Matrix (-ces)<br>• How to use the device (following the directions for use)<br>• When using the device (conditions of use)<br>• the person using the device (operator or target user)<br>• what for (goal condition)<br>• Who (target population) the device is being used for
**Reference Standard**	- Clinical reference standards for regulatory purposes are the best method available to establish the true condition of subjects<br>- Clinical reference standards should be established before starting the study, and appropriate standards should be used and interpreted as they are updated with technological advances
**Subject selection and sample collection**	- Subject preparation, specimen collection, storage, and handling procedures are important components that must be fully described in the study protocol<br>- In a prospective study design, a pre-specified protocol is used. These protocols pre-specify the study design, including inclusion/exclusion criteria, subject recruitment and selection methods, testing protocols, and analysis methods<br>- In a retrospective study design, the researcher searches for subjects through available data, specimens, images, or other information stored on media or devices<br>- This includes going to tertiary care centers to obtain samples or using enrollment data from previous studies with long-term follow-up
**Blinding**	- Results from clinical reference standards or other diagnostic assessments should not be recognized and should be blinded, as multiple assessments and users/readers may be involved
**Researcher's proficiency**	- Skills through work training, research knowledge, and experience in reading results may affect the performance of the device.<br>- Therefore, the clinical research protocol should consider the performance variability according to the skill level of the researcher handling the device, and additional performance research by other researchers may be required.<br>- Sponsors may be required to document researcher training.

Table S5. Intended use and performance indicator of CDx

Intended use	Performance indicator	Subject of study	Type of study
**Treatment stratification**	Patient outcome measurements and interaction analysis	all new entrants (All patients receiving drug therapy)	Prospective randomized and retrospective trials, correlational trials
**Treatment selection**		Biomarker positive patients	

Table S6. Performance evaluation according to the IVDR

IVDR Annex 13. Part A 1. Performance evaluation	
**Plan**	- Specifications for the intended purpose of the device (specification)<br>- Equipment characteristics (Annex 1. Chapter 2, Section 9, Chapter 3, Section 20.4.1 (c))<br>- Specifications of the analyte or marker determined by the device<br>- Specifications for the purpose of the device (specification)<br>- Identification of certified reference materials or standard measurement procedures to enable quantitative traceability<br>- Clear identification of specific target patient populations with clear symptoms, limitations and contraindications to use (specific drugs or treatments)<br>- Annex 1. Identification of general safety and performance requirements specified in Sections 1 to 9 (based on relevant scientific justification and analytical/clinical performance data)<br>- Description of methods used to assess analytical/clinical performance, limitations of the device, and description of accompanying information.<br>- Description of state-of-the-art technology (including relevant standards, CS, guidelines or best regulatory documents)<br>- Indication and description of the parameters used to determine the acceptability of the risk-benefit ratio for the intended use of the device and for its analytical/clinical performance, based on the latest knowledge in the field of medicine<br>- Identification and description of reference databases and other data sources used as a basis for decision-making, for software qualifying as a device<br>- Outline of the different stages of development, including indication of key timelines and description of possible acceptance criteria (including sequence and means of determining scientific validity, analytical/clinical performance)<br>- PMPF plan specified in Annex Part B
**Scientific validity**	- Relevant information on the scientific justification of devices measuring the same analyte or marker<br>- Scientific (peer reviewed) literature<br>- Relevant professional associations, unanimously agreed expert opinions/positions<br>- Results of proof-of-concept studies<br>- Results of clinical performance studies
**Analytical performance**	- As a general rule, always validated on the basis of analytical performance studies<br>- In the absence of a comparative method, other approaches may be used if proven appropriate<br>- In the absence of an approach, clinical performance studies comparing the performance of new devices with the latest clinical regulations are required
**Clinical performance**	- Proven based on one or more of clinical performance studies, scientific (peer reviewed) literature, and published experience from routine diagnostic testing<br>- Unless justification for reliance on other sources of clinical performance data is provided<br>- Proven and documented in clinical performance reports

Table S7. Clinical performance study according to the IVDR

IVDR Annex 13. Part A 2. Clinical performance studies	
**Purpose**	- Establish or verify performance of a device that cannot be judged by experience gained through analytical performance studies, literature, and routine diagnostic testing<br>- Used to demonstrate compliance with general safety and performance requirements related to clinical performance<br>- Data obtained from conducting clinical performance studies should be used in the performance evaluation process and should be part of the clinical evidence of the device
**Ethical considerations**	- Each phase of the clinical performance study is conducted in accordance with recognized ethical principles
**Design type**	- Designed in such a way as to maximize the relevance of data while minimizing bias
**Plan**	- Should be conducted based on the Clinical Performance Study Plan (CPSP) and should define the rationale, objectives, design and proposed analysis, methods, monitoring, conduct and record keeping<br>- Include the following information<br>: Single identification number<br>: Identification number of the client (name of the client, address of the registered office, contact information, etc.)<br>: Information about the researcher (contact information, research institute information)<br>: Study start date and planned duration<br>: Device identification information and description, use, analyte or label, quantitative traceability, manufacturer<br>: Information on the type of specimen being studied<br>: Overall summary of the study, design type, research objective, hypotheses, diagnosis and reference to state-of-the-art technology in medicine<br>: Expected risks and benefits of the device, description of the study, exceptions to studies using residual specimens, related medical procedures and patient management<br>: Instructions for use of the device, test protocols, training and experience required by the user, appropriate calibration procedures and controls, indications of other devices, medical devices, pharmaceuticals, or other items to be included or excluded, and to other comparable devices or methods used as references. description of<br>: Description of study design and validity, scientific robustness, detailed description of measures to minimize bias, and management of potential confounding factors<br>: Analytical performance, justification for omissions<br>: Clinical performance parameters, justification for any omissions, exceptions to studies using residual samples, specified clinical outcome/endpoints (primary/secondary) and their validity, potential impact on personal health and public health management decisions<br>: Population information (subjects, selection criteria, population size, vulnerable subjects, etc.)<br>: Information on the use of data from residual samples, gene or tissue banks, patient or disease registries, etc., description of reliability, representativeness and statistical analysis approaches, assurance of methods for determining the clinical veracity of patient samples.<br>: Monitoring plan

	: Data Management<br>: Decision algorithm<br>: Policy on CPSP Modifications, Deviations from CPSP, and Clear Prohibition of Waiver<br>: Responsibility for explaining the device (management of access to the device, follow-up, recovery of the device)<br>: Statement of Commitment to Compliance with Ethical Principles, Good Clinical Practice Principles, and Applicable Regulatory Requirements<br>: Description of the informed consent process (consent form)<br>: Safety Recording and Reporting Procedures<br>: Criteria and Procedures for Suspension or Early Termination<br>: Criteria and procedures for follow-up of subjects after completion, discontinuation or early termination, follow-up procedures for subjects who withdrew informed consent, and procedures for subjects who did not receive follow-up<br>: Procedure for delivering test results to the outside<br>: Policies related to reporting and presentation of results in accordance with legal requirements and ethical principles<br>: List of technical/functional features of the device<br>: References
**Report**	- Include documented information about the protocol plan, results and conclusions, along with negative findings, in a report signed by a physician or other authorized person<br>- Transparent, unbiased, clinically relevant results and conclusions<br>- Include enough information that an independent party can understand it without consulting other documents<br>- Include changes or non-compliance with protocols and data exclusions where appropriate

Table S8. Analytical and clinical performance evaluation indicators

Analytical performance	Clinical performance
Accuracy<br>Precision<br>Specificity<br>Measuring range and linear interval, such as quantitation range or detection limit<br>Analytical cutoff<br>Standard material<br>Information on the sample being collected<br>Possibility of occurrence of non-specific reactions and methods of suppression<br>Possibility of contamination and methods of elimination	Positive or negative consistency between the applied product and the licensed product or existing test method<br>Drug responsiveness using IVD-CDx

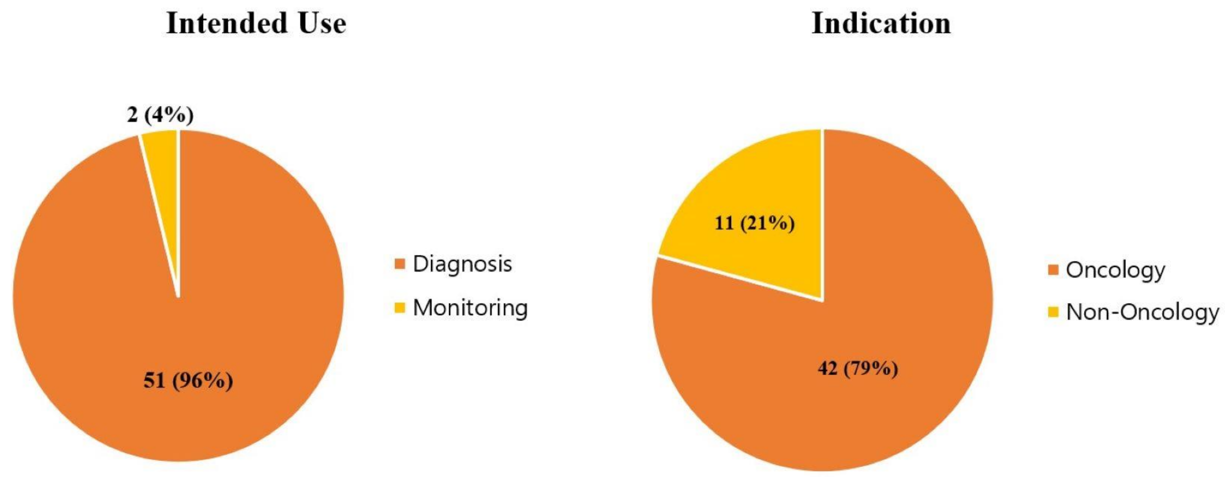

Figure S1. Approval status in FDA according to intended use and indication type. (a): Intended use. (b): Indication type. These graphs are analysis of CDx products approved by the FDA based on intended use and indication type. As a result of analyzing the intended use according to the definition of the device, 96% (51/53) were used to identify patients through disease diagnosis and 4% (2/53) were used to monitor a disease. Depending on the type of indication, 79% (42/53) appeared as a device for diagnosing cancer or tumor.

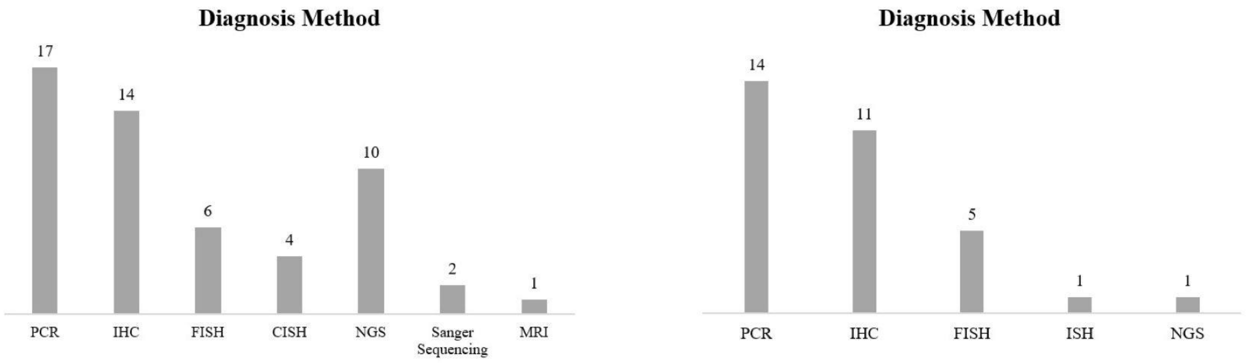

Figure S2. Approval status according to the diagnosis method. (a): FDA. (b): MFDS. These graphs are analysis of CDx products approved by the FDA and MFDS based on diagnosis method. Polymerase chain reaction (PCR) technology (32%) was applied the most frequently as the diagnosis method in FDA. All products in MFDS were used to identify patients by diagnosing cancer of oncology. In Particular, PCR technology (44%) was the most frequently applied diagnosis method.
