# Supplementary material for: Necessity of strengthening the current clinical regulatory for companion diagnostics: An institutional comparison of the FDA, EMA, and MFDS
Source: Mol Ther Methods Clin Dev. 2023 Aug 16;30:447–58. doi: 10.1016/j.omtm.2023.08.008 (PMC10474566; doi:10.1016/j.omtm.2023.08.008)
Supplement: Document S2. Article plus supplemental information [file mmc3.pdf]

Molecular Therapy
Methods & Clinical Development
Review

Necessity of strengthening the current clinical regulatory for companion diagnostics: An institutional comparison of the FDA, EMA, and MFDS

Su Lim Kang,[1] Jae Hyun Woo,[2] Na Hyeon Kim,[3] Ji Yean Kwon,[1,2,3] and Sung Min Kim[1,2,3]

[1]Department of Medical Device Industry, Dongguk University-Seoul, 26, Pil-dong 3-ga, Jung-gu, Seoul 04620, Republic of Korea; [2]Department of Regulatory Science for Bio-Health Medical Device, Dongguk University-Seoul, 26, Pil-dong 3-ga, Jung-gu, Seoul 04620, Republic of Korea; [3]Department of Biomedical Engineering, Dongguk University-Seoul, 26, Pil-dong 3-ga, Jung-gu, Seoul 04620, Republic of Korea

Companion diagnostics (CDx), which is essential in precision medicine, is changing to a personalized treatment approach. CDx is a test that identifies patients who can benefit from a specific drug and those who experience side effects of drugs for safe and effective treatment. Conversely, when CDx has inadequate diagnostic performance or has not been adequately validated in a particular treatment, treatment prediction based on diagnostic results is not possible. Given the importance of CDx for the clinical use of biomarkers, strict regulation is essential. Regulators are providing more stringent regulations and are developing or revising guidelines. For example, the EU's In Vitro Diagnostic Regulation has defined CDx for the first time, raising awareness of the importance of CDx. However, if a new clinical performance test needs to be performed to meet the latest specifications or requirements for clinical data, problems such as securing clinical samples or institutions, cost, and time may occur. Therefore, an efficient clinical regulatory process may be required to meet stringent regulatory requirements. This study examines the need to strengthen the current clinical regulatory framework for CDx through an institutional comparison of regulatory agencies (FDA, EMA, and MFDS).

INTRODUCTION

Precision medicine is changing the treatment of many diseases through targeted testing and treatment approaches. Companion diagnostics (CDx) based on biomarkers is essential for individualized treatment.[1] CDx pre-selects patients suitable for a targeted therapy. It also detects specific biomarkers to identify patients who might benefit from a particular drug and those who might be at risk of experiencing side effects.[2] Thus, patients expect better effects through personalized medical care. Accordingly, the demand for CDx is gradually increasing.[3]

Treatment prediction based on CDx results is not possible if the diagnostic performance is inadequate or the test has not been adequately validated for a particular treatment. The strict regulation of CDx is essential, as clinical uses of biomarkers are important.[4] CDx was recalled or removed from the market in 2019 due to inaccuracies in diagnostic performance. The United States Food and Drug Administration (FDA) recalled a CDx (FoundationOne CDx) used to identify colorectal cancer patients eligible for treatment with target drugs because of concerns about potential false-positive MSI-H (microsatellite instability marker—high).[5]

As the number of CDx grows, regulatory agencies are proposing tougher regulations and guidelines. The FDA has strengthened its leading position in providing a regulatory environment for CDx since the first approved CDx.[6,7] In particular, the definition of CDx was established in guidelines published in 2014, and guidelines for co-development and labeling are consistently presented. The newly presented In Vitro Diagnostic Regulation (IVDR) in the European Union (EU) has established strengthened clinical and regulatory requirements. It directly refers to regulatory requirements for CDx. The clinical regulation of CDx in the Ministry of Food and Drug Safety (MFDS) of Korea emerged with the development of guidelines in 2015. In 2018, separate guidelines for safety, performance, and protocol evaluation, different from the 2015 guidelines, were presented.

Various studies have been conducted on the clinical regulation of CDx. Studies examining the requirements of clinical regulations of CDx and the challenges of clinical implementation increased from 2014 to 2020 after the definition of CDx was first established by the FDA. A study in 2014 by Olsen and Jørgensen considered the scientific and medical challenges of CDx in achieving a high level of analytical and clinical validity, with a focus on regulatory requirements in many parts of the world.[8] In 2015 Lee and Shen described the regulatory model for CDx in the United States and discussed key strategies for a successful co-development program, changes in clinical management as technology advances, and regulatory considerations.[9] Recent studies have reviewed and updated new or existing regulations on the European side according to the application of the IVDR. Enzmann

https://doi.org/10.1016/j.omtm.2023.08.008.

Correspondence: Sung Min Kim, Ph.D., Department of Medical Device Industry, Dongguk University-Seoul, 26, Pil-dong 3-ga, Jung-gu, Seoul 04620, Republic of Korea.
E-mail: smkim@dongguk.edu

This is an open access article under the CC BY license (http://creativecommons.org/licenses/by/4.0/).

et al. (2016) reviewed potential problems caused by the new EU regulations, suggesting the importance of a close relationship and interface between pharmaceuticals and CDx.[10] Studies have also been conducted to consider the challenges of clinical implementation faced by CDx due to regulatory tightening[1] and discuss their impact.[11] However, when a new clinical performance test needs to be conducted to meet the latest specifications or requirements by the FDA and MFDS and recertified according to IVDR in EU, problems such as securing clinical samples or institutions, cost, and time taken for clinical trials may occur.[12] An efficient regulatory framework may also be required to address the challenges arising from stringent regulatory requirements. Although existing studies have reviewed regulatory changes and discussed challenges posed by strict clinical regulation, no studies have conducted institutional comparisons by country (FDA, European Medicines Agency [EMA], and MFDS) on the need to strengthen the process or framework for efficient clinical regulation.

Thus, the objective of this study was to review the need to strengthen the current clinical regulatory framework for CDx by comparing the requirements of regulatory agencies (FDA, EMA, and MFDS).

LITERATURE SELECTION AND DATA COLLECTION

Approval status of CDx products was analyzed in the databases of regulatory agencies by country to February 2023.[13,14] Europe was excluded from the search because of restrictions on access due to the transparency of approval information at the notified bodies, responsible for approving CDx in the EU. Guidelines published by regulatory agencies by country (FDA, EMA, and MFDS) were used for institutional comparisons. Although this study focused more on analyzing the guidelines, which included the contents of CDx clinical regulations, reports and seminar data were also reviewed for a broader analysis from various perspectives, such as that of industry.

DATA EXTRACTION AND ANALYSIS

Diagnostic name, manufacturer, indications, diagnosis method, description, and intended use were extracted from the collected list to analyze approval status. Only performance (analytical and clinical performance) and usefulness (clinical trial) evaluation methods to secure the safety and efficacy of CDx were extracted in the institutional analysis of regulatory agencies (FDA, EMA, and MFDS). Content on analysis and clinical verification, or clinical efficacy and significance, were extracted from the guidelines related to clinical regulations for institutional comparisons.

The total quantity of CDx products approved by the FDA and MFDS, the intended use, and the proportion according to indication type and diagnosis method were included in the approval status analysis. Differences between institutions were analyzed according to performance evaluation and clinical trial. Based on the findings, an SWOT (strengths, weaknesses, opportunities, and threats) analysis was conducted to review the need for strengthening the current clinical regulatory framework for CDx. The overall research model is shown in Figure 1.

OVERALL ANALYSIS

A total of ten (four FDA, three EMA, and three MFDS) regulations and guidelines were analyzed in the overall analysis of CDx by country.[15–24] The FDA published the "In Vitro Companion Diagnostic Devices" guideline in 2014, establishing the definition of CDx for the first time. CDx was defined as: an *in vitro* diagnostic (IVD) companion device that could be essential for the safe and effective use of a corresponding therapeutic product (identify patients who are most likely to benefit, be at increased risk for serious side effects as a result of treatment with a particular therapeutic product, and monitor response to treatment to achieve improved safety or effectiveness). The FDA categorizes devices into three classes (1, 2, and 3) based on the risk that might be assigned to the patient or user. Most class 1 devices are exempt from pre-market notification (510(k)) requirements, class 2 devices follow 510(k), and class 3 devices follow pre-market approval (PMA). CDx is class 2 or 3, and follows the 510(k) or PMA pathway under the federal Food, Drug, and Cosmetic Act. In particular, there are differences in the regulatory pathway depending on whether a new drug or device is approved, and the evaluation of technical documentation also depends on the approval pathway (510(k) or PMA). With the introduction of the IVDR, the EMA newly established the definition of CDx as a device that is essential for the safe and effective use of a corresponding medicinal product (identify patients who are most likely to benefit and to be at increased risk of serious adverse reactions as a result of treatment). Definitions of CDx established by the FDA and EMA show a clear difference. The FDA includes "devices that monitor response to treatment" in the definition of EMA. The IVDR assigns devices to four risk classes (A, B, C, and D) based on patient and public health risks. EMA classifies CDx as class C according to rule 3, and requires independent conformity assessment and technical document evaluation for the CDx. The MFDS generally follows regulations similar to those of the FDA and defines a CDx as a test system that performs tests that predict the responsiveness and safety of a drug for use in the treatment of a specific patient (selection of patients who can expect a high effect or risk of serious side effects by specific treatment, determining the dosage, timing of administration, and discontinuation of administration, and selecting patients from a group that has been confirmed to be effective through sufficient research). The MFDS classifies IVDs into four classes (1, 2, 3, and 4) according to the degree of potential risk to individuals and public health. CDx are categorized as class 2 or 3, and the regulatory path varies depending on the development path (new or existing approval) (Table 1).

ANALYSIS OF APPROVAL STATUS

We identified CDx approved by the FDA and MFDS. A total of 53 and 32 products have been approved by the FDA and MFDS, respectively. As a result of the analysis, various types of CDx were identified according to intended use, indications, and diagnosis method. Detailed analysis results are presented in the supplemental information (Tables S1 and S2; Figures S1 and S2).

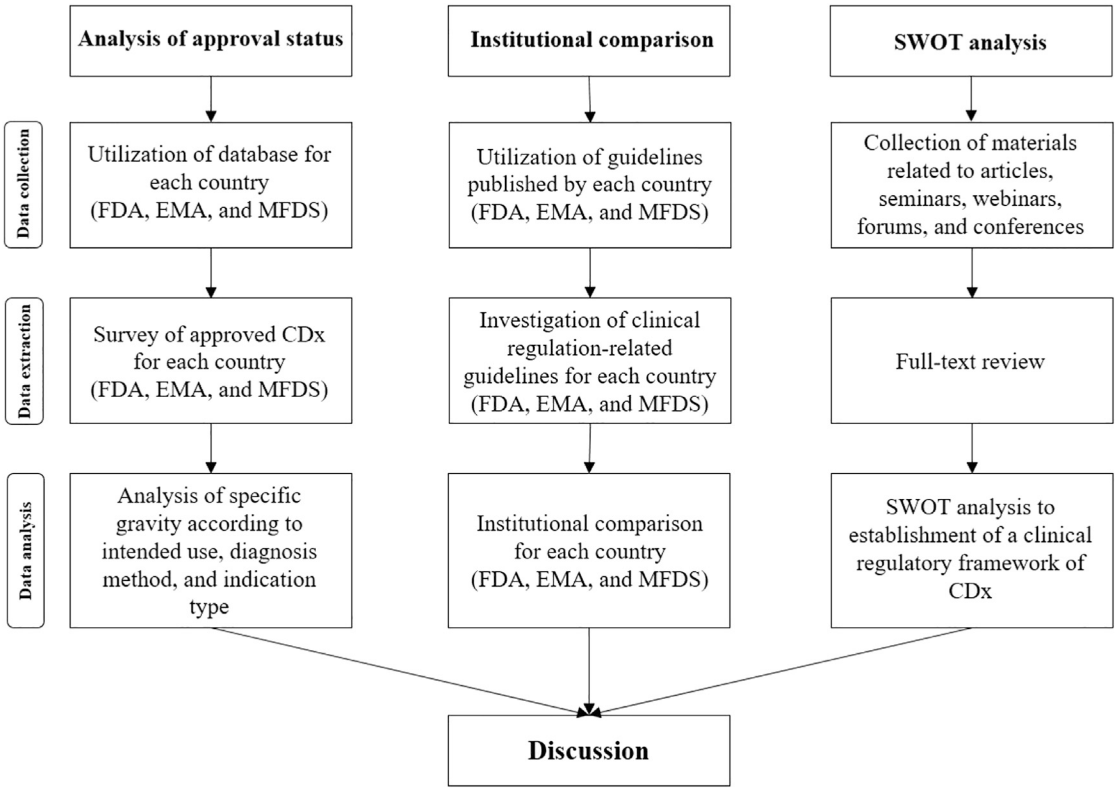

Figure 1. Research model
This flowchart presents the process of analysis of approval status, institutional comparison, and SWOT analysis.

INSTITUTIONAL ANALYSIS

United States (FDA)

The FDA considers intended use, the selection of an appropriate study population, and the minimization of specific sources of bias as important factors influencing the design of clinical investigations of diagnostic devices (Table S3). Diagnostic clinical performance is evaluated by a measure that quantifies how closely the output of a diagnostic device correlates with the clinical reference standard used to evaluate patients for that purpose. The selection of appropriate clinical performance measures will depend on the device's intended use, characteristics, and clinical reference standard, and the study goal is set to establish the diagnostic clinical performance of the device to support a risk-benefit analysis. Performance evaluation of a CDx can be achieved by evaluating analytical performance, clinical performance, and method comparison.[25]

A well-controlled clinical investigation is essential for demonstrating the scientific evidence and effectiveness of a device. According to Code of Federal Regulations title 21 (21 CFR) 860.7, valid scientific evidence is evidence of well-controlled investigations, partially controlled studies, unmatched controlled studies and objective trials, well-documented case histories conducted by qualified professionals, and significant human experience reports for marketed devices.[26] Where appropriate, the type of evidence that may be needed to determine the reasonable assurance that a device is safe includes investigations using laboratory animals, human subject investigations, nonclinical studies, and analytical studies of IVDs. A device can be demonstrated to be effective if, on the basis of valid scientific evidence, it can be determined that it is applied for its intended use and conditions of use for a significant portion of the target population. The plan, protocol, and results of the clinical study should include: the scientific rationale for the study, population definition (including selection and exclusion criteria), the intended use, endpoint, protocol, and outcome analysis method (statistical analysis, including data analysis) (Table S4). Verification of such a CDx should include procedures from sample preparation to the reporting of results.[9]

Europe (EMA)

Regulations on clinical evidence were strengthened when Europe transitioned from In Vitro Diagnostic Medical Device Directive (IVDD) regulations to the IVDR. Accordingly, the manufacturer must specifically describe and justify the level of clinical evidence and plan, and conduct and document the performance evaluation. Therefore, based on the latest knowledge in the medical field, scientific proof must be provided that the intended clinical benefit can be achieved and that the device is safe in accordance with the following three indicators.[27]

Table 1. Regulatory requirements for CDx by FDA, EMA, and MFDS

	FDA	EMA	MFDS
Definition	*in vitro* diagnostic devices that provide essential information for the safe and effective use of the treatment<br>- identify patients who are most likely to benefit from a particular therapeutic product<br>- identify patients likely to be at increased risk for serious side effects as a result of treatment with a particular therapeutic product<br>- monitor response to treatment with a particular therapeutic product for the purpose of adjusting treatment to achieve improved safety or effectiveness	a device which is essential for the safe and effective use of a corresponding medicinal product<br>- identify, before and/or during treatment, patients who are most likely to benefit from the corresponding medicinal product<br>- identify, before and/or during treatment, patients likely to be at increased risk of serious adverse reactions as a result of treatment with the corresponding medicinal product	a test system that performs a test to predict the reactivity and safety of a drug in order to use it for the treatment of a specific patient<br>- selection of patients who can expect a high effect on a specific treatment<br>- selection of patients at risk of serious side effects by specific treatment<br>- determining the dosage, timing of administration, and discontinuation of administration for a specific treatment in a specific patient<br>- select patients from a group that has been confirmed to be effective through sufficient research on the treatment itself, even though information on the safety and effectiveness of the treatment is lacking in other groups
Class	class 2 or 3	class C	class 2 or 3
Regulatory path	review and approval under the Federal Food, Drug, and Cosmetic Act (FD&C Act) and related medical device regulations<br>(1) for new therapeutics, verifying that the device has been adequately validated and meets applicable standards for safety and effectiveness or substantial equivalence for the intended use indicated in the therapeutic product's labeling<br>(2) approval of therapies without an approved IVD device<br>- new therapies to treat serious or life-threatening conditions<br>: may decide to approve a therapeutic product if it is intended to treat a serious or life-threatening condition for which no satisfactory alternative treatment exists and for which there is a benefit to using an IVD<br>- already approved treatment<br>: in general, supplementation of previously licensed treatments is not permitted until the device is approved, but change approval is possible if it is judged that there is a risk due to lack of device	notified bodies shall apply for all devices the technical documentation assessment procedure described in section 5.2 of annex IX, the technical documentation assessment procedure described in and in accordance with directive 2001/83/EC of the European Parliament and of the Council, and consult with the competent authority designated by the member state or with the EMA in accordance with section 5.2 of annex IX	- in the case a new IVD-CDx is developed, the drug is reviewed by the Drug (bio-herbal drug) Review Department in accordance with "Regulations on product approval, notification, and review of pharmaceutical products" or "Regulations on product approval and review of biological products" for drug efficacy in the selected patient group. Companion diagnostic medical devices are reviewed by the Medical Device Review Department in accordance with the "Regulations on medical device approval, notification, review"<br>- if a product equivalent to an already licensed companion diagnostic medical device is developed, review clinical trial data comparing the equivalence between the already licensed product and the new product

(*Continued on next page*)

Table 1. *Continued*

	FDA	EMA	MFDS
Evaluation of technical documentation	class III medical devices require PMA procedure under section 515 of the FD&C Act<br>- administrative and limited scientific review by FDA staff to determine completeness (review of acceptance and submission)<br>- in-depth scientific, regulatory and quality system review (substantive review) by appropriate FDA staff<br>- review by an appropriate advisory board (panel review)<br>- final review, documentation, and notification of FDA decisions<br>– > a New Drug Application for a treatment is submitted and reviewed by Center for Devices and Radiological Health while being reviewed by Center for Drug Evaluation and Research or Center for Biologics Evaluation and Research<br>class II medical devices require a 510(k) procedure under section 213 of the FD&C Act<br>- prove that it is substantially equivalent to a legally marketed device<br>- same purpose + same technical characteristics<br>- same purpose + different technical characteristics, but no problem in safety/efficacy + proven to be as safe and effective as licensed devices<br>– > review the scientific methods used to evaluate differences in technical characteristics and performance data to determine whether the device is as safe and effective as its predecessor	- the companion diagnostic device manufacturer must submit an application for evaluation of technical documents to the certification body<br>- the application must be able to assess compliance with the relevant requirements to understand the characteristics and performance of the device. In particular, it should be possible to assess the suitability of devices related to medicinal products<br>- prior to issuing EU technical documentation evaluation certificates for companion diagnostic devices based on the draft safety and performance summary and the draft instructions for use, the notified body shall, in accordance with directive 2001/83/EC, consult either one of the competent authorities designated by the member state or the EMA. You should seek a scientific opinion on the suitability of the device in relation to the medicinal product<br>- the pharmaceutical consultative authority shall provide its opinion to the certification body within 60 days from the date of receipt of all necessary documents<br>- the notified body must fully consider scientific opinions when making decisions, and must deliver the final decision to the pharmaceutical consultative authority<br>- establishment of Community Procedures and establishment of European Medicines Agency for Authorization and Supervision of Pharmaceuticals for Human and Veterinary Use<br>- before making any changes that affect the performance and intended use of the device or the suitability of the device for medicinal products, the manufacturer shall inform the notified body of such changes. The Pharmaceuticals Consulting Authority shall provide its opinion within 30 days of receipt of all necessary documents relating to the change	- a person who wants to be reviewed for technical documents, etc. must fill out the “request for review of medical device technical documents, etc.” according to the form in annex 7 of the Enforcement Rule<br>- submit the attached data along with an electronic recording medium (CD) created with a dedicated program determined by the Minister of Food and Drug Safety<br>- if some of the attached data is unnecessary due to the nature of the product, the reason must be stated in detail<br>- foreign data must be accompanied by a summary in Korean and original text extracting key points, and translations can be requested only if necessary<br>- an essential equivalence comparison table compared with products that have already been licensed/certified must be submitted and attached<br>- if any of the following requirements apply, data on clinical performance tests must be submitted<br>: class 4 *in vitro* diagnostic medical device test<br>: high-risk test of the method of collecting samples from the human body<br>: a test in which the results of a clinical performance test cannot be confirmed with an already established medical diagnosis method or an approved/certified *in vitro* diagnostic medical device<br>: a test to diagnose together with medicines (limited to tests on devices that are not equivalent to medical devices that have already been licensed/certified and the purpose of use, principle of action)

- Scientific validity: how good is the evidence for the associations between biomarkers and clinical or physiological conditions?
- Analytical performance: how well does the device detect the biomarker?
- Clinical performance: how good is the device at selecting patients who will respond to the drug?

Scientific validity is supported by studies evaluating analytes for potential clinical applications in the international medical device regulators forum. Analytical performance evaluates the probability of how well a device detects a biomarker. Clinical performance has a different goal from that of analytical performance. Analytical performance research focuses on the analysis target, and clinical performance research focuses on the patient. There are two options for clinical performance data.

- Clinical performance defined as a correlation with a clinical condition or disease: clinical performance data and reports are required for devices that measure a specific analyte that is associated with a clinical condition or disease and has a medical decision point (cutoff).
- Clinical performance defined as a correlation with a physiological or pathophysiological state: analyte measurements that do not have a clear medical decision point (cutoff) or those that are not related to clinical status may be defined as being correlated with physiological or pathophysiological conditions, or justification for missing clinical performance data may be considered.

Korea (MFDS)

The MFDS applies a risk-based approach to review the intended use (whether it provides reasonable certainty about safety and effectiveness) and the level of risk resulting from a false-positive or false-negative result. The evaluation is based on the premise that when an *in vitro* companion diagnostic device (IVD-CDx; the English name in MFDS) uses biomarkers to select patients for drug administration, the safety, efficacy, and efficiency of the drug are directly affected by the performance of the device. Therefore, evaluations are performed four ways: evaluation of the IVD-CDx in pharmaceutical clinical trials, evaluation of IVD-CDx equivalence, and analytical and clinical performance evaluation. Clinical significance and clinical cutoff values for an IVD-CDx should be established and evaluated using the results of pharmaceutical clinical trials conducted on patients classified according to IVD-based tests. Therefore, when applying for permission, an explanation of the clinical trial results of the relevant drug, including information on the name of the drug used in the clinical trial, test name, test method, and summary of the test results, should be provided.

Institutional comparison

Clinical validation requirements for CDx in the United States, Europe, and Korea have a common goal of ensuring CDx accuracy and reliability. In addition, for the purpose of performance evaluation of CDx by country (FDA, EMA, and MFDS), there is a commonality in that the purpose of use is important to secure safety and effectiveness. However, differences in evaluation method exist because of the unique requirements of each regulatory agency. In particular, FDA values associations between product characteristics and clinical reference standards, while MFDS values risk-based evidence collection for safety and efficacy.

In analytical performance, the FDA specifies that it should consider the type of studies needed to support marketing approval of the device and be planned accordingly. It is important that adequate samples are collected in sufficient quantities, banked (if analyte stability permits), and properly maintained to support the full range of analytical studies. Collecting appropriate patient characteristics and pathological characteristics of the sample (e.g., tumor content, necrosis, obesity, presence of large amounts of epilepsy, and other characteristics) can help support conclusions about assay performance. Parameters such as sensitivity, specificity, reproducibility, precision, and accuracy should be established to evaluate analytical performance, and the stability of samples and reagents should be evaluated. The EMA judges assay performance as the basis for conducting clinical trials. Analytical performance should collect appropriate criteria for sample collection and the handling or control of endogenous and exogenous interferences and cross-reactivity, including analytical sensitivity, analytical specificity, precision (repeatability and reproducibility), accuracy, limits of detection and quantification, measurement range, linearity, and bias. In the EMA, reports of the new requirements for providing clinical evidence (scientific validity, analytical performance, and clinical performance) are mandatory. Therefore, the manufacturer must demonstrate the conformity of the device to general safety and performance requirements by evaluating all relevant scientific validation, analytical data, and clinical performance data. Clinical evidence must document the following items in the performance evaluation report: justification of the approach taken to collect clinical evidence, literature search methodology, and literature search protocol or report; the technology or device on which the device is based or its intended purpose and the performance or safety claims of the device; scientific validity and the nature and extent of the evaluated analytical and clinical performance data; clinical evidence as acceptable performance for state-of-the-art technology in medicine; post-market performance follow-up and any new conclusions derived from the report. In particular, performance evaluation reports for class C devices should be updated with data at least annually, if necessary. The performance evaluation report shall be part of the technical documentation. Demonstration of scientific validity and analytical and clinical performance should be assessed by identifying unresolved issues or gaps in the data and reviewing available data related to test devices and their intended use by a systematic scientific literature review and an assessment of the safety and performance of the device conducted by evaluating all relevant data and generating new or additional data needed to resolve outstanding issues. The MFDS evaluates whether there is a change in the user or environment to confirm the reliability of the product itself. Evaluation indicators include accuracy, precision, specificity, measurement of quantification range or detection limit, point and linearity interval, analytical cutoff, standard material, sample information, interference reaction, and possibility of contamination.

Regarding clinical performance, all agencies specify that devices should be evaluated to assess the adequacy of selecting eligible patients. The FDA specifies that devices should be evaluated when the device type and intended use are new, and new clinical data are required for new analytes, new indications, and new methodologies. In particular, clinical diagnostic accuracy is important because CDx validation must demonstrate the ability to predict treatment outcomes for individual patients. Therefore, clinical sensitivity, specificity, and positive and negative predictive values are important indicators. Method comparisons generally compare the performance of a new device with an existing device, and should provide information such as study design, population, sample size settings, sample type, criteria for validity, and protocol. According to FDA guideline 2013, clinical validation requires collecting evidence with drug test data and evaluating progression-free survival, objective response rate, and overall survival.[7,15] Manufacturers should consider designing clinical trials to support the evidence for both the treatment and the device. Clinical trials should consider different factors depending on where the treatment and device are co-developed (initial treatment development) and later treatment development. Prospective or retrospective study design types can be conducted for initial treatment development. However, in principle, they should be based on prospective clinical trials. In addition, an analysis cutoff (clinical decision above or below the test value) should be established to adequately define the population. The cutoff value is intended to indicate a point at which subjects suitable for randomization can be reliably identified, appropriate doses selected, or other clinical trial decisions made. Thus, a positive or negative point for the target marker should be specified. Specifying the cutoff before using the test in a clinical trial is a very important point because a change in the cutoff can change the patient classification (positively or negatively). The statistical analysis plan should include a sensitivity analysis for missing results and should compare the distribution of variables (characteristics of the sample, handling, and treatment) that may affect the results of the analysis. In particular, the impact of missing data on clinical performance (e.g., hazard ratios for subsets of marker definitions) should be analyzed. In late-stage therapy development, if a clinical trial is properly designed for a population based on marker detection, the results can be used to establish the clinical effectiveness of the device. The appropriate clinical trial design will depend on the intended use of the device and the predictive or prognostic properties of the markers, but may include factors such as the intrinsic properties of the markers (predictive, prognostic, or useful), disease progression, safety profile, and negative population (potential benefit). According to FDA guideline 2016, to prove that the results of the "training set (candidate CDx)" sample used to establish the clinical decision point and analysis criteria are very similar to the clinical trial assay (CTA), a "bridging study" between the two tests can be conducted by utilizing the original sample and a pre-specified statistical analysis plan. This evaluates the concordance between the two tests using the same sample of eligible patients to evaluate the effectiveness of the treatment in patients whose marker status was determined by the candidate CDx. High analytical concordance with the CTA indicates the high performance of a candidate CDx in predicting the effectiveness of a therapeutic agent.[18] According to the EMA, clinical performance must be demonstrated and maintained throughout the lifetime of the device as specified by the manufacturer, including diagnostic sensitivity, diagnostic specificity, negative or positive predictive values, likelihood ratio, and expected values in normal and affected populations. The indicators of clinical performance are highly dependent on the intended purpose and, in the case of a CDx, the purpose is divided into treatment stratification (predicting treatment response) and treatment selection (intended use of the selected CDx) (Table S5). Performance evaluation of a device is a continuous process of evaluation and analysis to demonstrate the scientific validity and the analytical and clinical performance of the device as stated by the manufacturer with respect to its intended use. Evaluations must be thorough and objective, taking into account both favorable and unfavorable data. The intended use underlying the performance evaluation plan is the sole responsibility of the manufacturer and directly determines the level of performance studies and post-market performance follow-up activities. If the use of other sources of clinical performance data cannot be justified, clinical performance studies must be conducted. Article 57 of IVDR requires the manufacturers to ensure that devices used for performance studies comply with the general safety and performance requirements described in annex 1, in addition to those covered by performance studies (Table S6). Where appropriate, performance studies should be conducted under conditions similar to the normal use conditions of the device and should be designed and conducted in a manner that ensures that the clinical data generated are scientifically valid and reliable. CDx studies should consider the intended purpose, medical treatment, and patient outcomes and should conduct clinical performance studies to demonstrate clinical effectiveness (Table S7). The EMA specifies that a clinical development program must be conducted together with a drug if a CDx is co-developed with the drug and gets market approval for the first time or if there is a change in the indication. A bridging study in which the device evaluates the pivotal clinical trial of the drug or the conformity of the device used in the pivotal clinical trial of the drug may also be performed. If a bridging study is performed, it should provide a sufficient basis for concluding that its performance is comparable to that of the device used in clinical trials of the drug. Consequently, the safety and effectiveness of the drug should be demonstrated to be identical to those described in the summary of the product. Clinical performance data may be substantiated based on one or a combination of clinical performance studies, scientific literature, or published experience from routine diagnostic testing. The demonstration of clinical performance can be direct, indirect, or a combination of both. Direct demonstration means that data are based on the specific device produced from a study using prospectively collected specimens or leftover specimens. Indirect demonstration means that data are based on a literature search or comparison with a reference device (method comparison). Direct demonstration yields a stronger level of evidence of clinical performance data than indirect demonstration. Therefore, it should be applied to higher risk classes or new devices. In particular, method comparison studies can provide evidence of equivalence to a reference device with strongly approved clinical evidence.[28] The follow-up CDx

is a device that pursues the same indication as the jointly developed CDx (original CDx). Although it targets the same biomarkers, it does not necessarily pursue the same technology as the original CDx nor does it run concurrently with clinical trials of drugs. Therefore, the follow-up CDx should be similar to the original CDx in terms of clinical evidence (analytical and clinical performance) and related safety and efficacy of the drug. To this end, sufficient documentation of the comparative analytical performance results and the clinical performance results for the safety and efficacy of the drug should be provided in the summary of product characteristics (SmPC).[20] The MFDS specifies that, if an IVD-CDx that is scheduled for approval application is not used in clinical pharmaceutical trials, an equivalence test (clinical trial) can be conducted between the IVD-CDx and an approved product used as the control. It is best to use samples collected from subjects who participated in clinical trials of related drugs when testing for IVD-CDx equivalence, but if it is difficult to use the samples of subjects included in the clinical trial, an equivalence test may be conducted separately using samples of a new group of subjects collected and preserved based on the same selection criteria as those of the clinical trial. Criteria, such as positive or negative matching rates required to determine an appropriate IVD-CDx, are evaluated by considering the characteristics of the target disease, the number of patients (realistically identifiable number of functional cases), and confidence intervals. Analytical and clinical performance evaluations should present verification indicators that can demonstrate the safety and effectiveness of an IVD-CDx (Table S8). Clinical performance (clinical sensitivity and specificity) should be evaluated using test data on human-derived specimens to prove the safety and efficacy of an IVD-CDx. Clinical performance should be performed to secure three pieces of evidence: evidence on the prognostic impact related to the status of the biomarker, evidence on the impact of the proposed test on the patient's health, and direct, high-quality evidence and statistical significance determinations obtained from patients. Therefore, a comparison of the effects of targeted therapy and general therapy may be required when an IVD-CDx is used to predict treatment effects. In addition, assessing the health effects of patients may require the consideration of random assignments and the morbidity of the test and control groups. If the cutoff value is set on the basis of the clinical cutoff value (sensitivity and specificity), a valid clinical rationale should be provided. In this case, data on test subjects (demographics, selection criteria, exclusion criteria, and the total number of patients) and statistical methods, such as receiver-operating characteristic curve analysis, should be presented. If it is difficult to evaluate clinical sensitivity and specificity because disease diagnosis is not the main purpose of the test, clinical data showing the clinical effectiveness of the test can be submitted. In the case of discrepant clinical sensitivity and specificity assessment results, the cause of the discrepancy should be investigated by conducting additional tests, using other methods or markers, reviewing the patient's clinical condition or diagnosis, and follow-up tests.[22–24]

While the FDA and EMA firmly specify individual evaluation indicators, the MFDS lacks information on specific indicators other than its emphasis on cutoffs. In clinical trials to establish clinical usefulness, the FDA and MFDS recommend the use of clinical trial data for drugs. The EMA may conduct study designs differently depending on whether the drug and CDx are co-developed. While the FDA and MFDS recommend joint development and accept both prospective and retrospective studies, the EMA presents recommendations for collecting evidence through other routes rather than recommending joint development (Table 2).

The institutional comparison is an important point that can support the need for an efficient regulation process. By identifying commonalities and differences in requirements, it can avoid duplication and streamline of regulation and consequently accelerate product approval. In particular, understanding the institutions unique to regulators can also access global markets. In addition, if it does not end with simply creating an efficient process based on regulatory comparison but leads to opportunities for international harmonization and standardization, it will contribute to improving the accessibility of infrastructure by understanding the regulatory environment of various countries.

SWOT ANALYSIS

An SWOT analysis is a strategic planning tool used to identify and analyze the strengths, weaknesses, opportunities, and threats of a particular situation or decision. Strength is analyzed as an internal positive factor. Opportunity is analyzed as an external positive factor. Weakness is analyzed as an internal negative factor. Threat is analyzed as an external negative factor. In this analysis, we reviewed full texts of articles and press releases published and announced by the institutes, working groups, or associations (Institute of Medical Device Safety Information, Medical Device Coordination Group, MedTech Europe, Korea Medical Devices Industry Association, among others).[12,25,29–40] An SWOT analysis of the need to strengthen the clinical regulatory framework is conducted as follows.

- Strengths: self-positive factors due to framework reinforcement.
- Weaknesses: internal negative factors due to framework reinforcement.
- Opportunity: positive factors in the global market due to strengthened framework.
- Threats: potential problems or negative factors in the global market due to framework reinforcement.

Strength works to ensure the accuracy and reliability of CDx to improve patient outcomes and can reduce healthcare costs. It can also aid in efficient clinical implementation by providing CDx developers with a clear process for the standards required for validation. In conclusion, it promotes collaboration among diagnostic companies, pharmaceutical companies, and regulatory agencies that could lead to more efficient and effective CDx development and approval. These strengths can be further highlighted by the opportunity that the demand for CDx is rapidly increasing as the paradigm shift in precision medicine enables personalized treatment. Accordingly, availability of accurate and reliable CDx tests are increasing to provide patients with more personalized and effective care. It can also be an important

Table 2. Institutional comparison by FDA, EMA, and MFDS

Division		FDA	EMA	MFDS
Performance evaluation	Measure	quantify the relationship between the output for the purpose of use of the device and the clinical reference standard	scientifically proven safety and achievement of intended clinical benefit of the device	evaluate the performance that is directly affected by the safety, effectiveness and efficiency of drugs
	Main factor	- intended use<br>- characteristics of the device<br>- clinical reference standard	- intended use	- intended use<br>- whether a reasonable certainty of safety and effectiveness is provided<br>- risk level according to false-positive or false-negative result
	Evaluation method	- analytical performance<br>· appropriate sample collection and storage/maintenance required<br>· collection of appropriate pathological characteristics for the sample<br>· evaluation indicators: sensitivity, specificity, reproducibility, precision, accuracy, stability of samples and reagents<br>- clinical performance<br>· conducted in case of new device type and intended use<br>· demonstrated ability to predict treatment outcome for individual patients<br>· evaluation indicators: sensitivity, specificity, positive and negative predictive value<br>- method comparison<br>· compare the performance of the new device to the existing device	- analytical performance<br>· evaluation of biomarker detection rate of the device<br>· basic data of clinical performance<br>· evaluation indicators: sensitivity, specificity, precision (repeatability, reproducibility), accuracy, limits of detection and quantification, measurement range, linearity, bias, interference response<br>- clinical performance<br>· assessing the adequacy of screening eligible patients<br>· depending on the correlation with condition/disease or physiological/pathophysiological condition, options differ<br>· evaluation indicators: sensitivity, specificity, positive and negative predictive value, likelihood ratio, expected value in normal and affected populations	- analytical performance<br>· evaluation indicators: accuracy, precision, specificity, measurement of quantification range or detection limit, point and linearity interval, analytical cutoff, standard material, sample information, interference reaction, possibility of contamination<br>- clinical performance<br>· securing evidence of prognosis, health effects, and statistical significance judgment<br>· provision of reasonable evidence for the establishment of the cutoff standard<br>· evaluation indicators: positive or negative consistency between applied products and licensed products or existing test methods, drug reactivity using IVD-CDx<br>- evaluation of equivalence of IVD-CDx<br>· conducted when IVD-CDx, which is scheduled to apply for approval, is not used in clinical trials of pharmaceuticals<br>· set the previously licensed product as a control group<br>· retrospective study available
Clinical trial		- evidence gathering with drug trial data<br>- presentation of evidence that can meet the purpose of use of the device<br>- conducting research (animal experiments, clinical trials, non-clinical trials and tests using *in vitro* diagnostic devices) to present valid scientific evidence<br>- conducting prospective or retrospective studies<br>- set cutoff for target marker<br>- analysis of missing data and impact assessment<br>- conducting bridging study to prove similarities with CTA<br>- evaluation indicators: progression-free survival period, objective response rate, overall survival	- in case of initial marketing approval or indication change of CDx co-developed with drugs<br>· conducting pivotal clinical trials of pharmaceuticals or bridging studies to evaluate conformity of devices<br>· confirmation that there is no effect on clinical performance that is incompatible with the safety and efficacy described in the SmPC of the drug<br>- in case of CDx development in late therapeutic product development<br>· pursuing indications with the same purpose as jointly developed CDx<br>· targeting the same biomarker, but not in parallel with clinical trials<br>· not necessarily based on the same technology as the original CDx<br>· analytical and clinical performance and the safety and efficacy of the related drug should be very similar to the original CDx	- IVD-CDx evaluation in pharmaceutical clinical trials<br>· establishing clinical significance and acceptance criteria using clinical trial results of pharmaceuticals<br>· description using the outline of clinical trial results of pharmaceuticals

opportunity aspect that countries around the world have announced various guidelines and regulations for CDx and continue to provide regulatory support. However, strengthening the regulatory framework can lead to time consumed due to huge data resources and lack of harmonization of specific standards and requirements, potentially delaying CDx and drug co-development as well as approval. In addition, the strengthening of these regulations may be challenging in countries or regions with limited regulatory capacity or resources, as not all countries have the necessary infrastructure in place. Limited healthcare resources in countries or regions potentially can limit patient access to CDx testing due to large disparities in access.

CONCLUSIONS

In this study, the need to establish a clinical regulatory framework for CDx was reviewed through institutional comparison of regulatory agencies, namely the FDA, EMA, and MFDS. In the analysis of the overall regulations regarding CDx by country, CDx was defined as a device that provides information through diagnosis for the safe and effective use of therapeutics. While the safety and effectiveness of a CDx is important, the safety and effectiveness of the treatment must also be secured for patients identified for CDx use. Therefore, the key to a CDx is to secure clinical usefulness (safety and effectiveness of drugs) together with clinical effectiveness (performance of the device). Since a CDx, by definition (providing essential information for the safe and effective use of medicines), affects clinical trials of drugs, it is subject to more complex regulations than other IVDs.

The analysis of the FDA and MFDS approval statuses of CDx confirmed that CDx with different purposes was developed and licensed for various diseases by applying various technologies. As the development of a CDx can be related to complex clinical regulations and processes, it is necessary to consider several variables such as disease-specific (oncology or non-oncology) and technology-specific (PCR, immunohistochemistry, fluorescent *in situ* hybridization, and next-generation sequencing) variables and the intended use (diagnosis, screening, prediction, and monitoring). One study suggested that a CDx requires case-by-case consideration because it has scientific, regulatory, and other unique characteristics given the diversity of devices.[2] Therefore, a large-scale case study is needed to prepare a guide for the efficient clinical implementation of various CDx.

There are some differences in the clinical validation requirements for CDx in the United States, Europe, and Korea. Although there is a common goal of ensuring accuracy and reliability based on the intended use of a CDx, each regulatory body has unique requirements. The FDA emphasizes the relevance of the final outcome values to the clinical reference standard, and the MFDS focuses on the level of risk by applying a risk-factor-based approach. Analytical performance and clinical performance are common evaluation indicators, and methods for comparison to existing devices (FDA method comparison, MFDS equivalence evaluation) are presented. In particular, the evaluation of clinical usefulness, which is valued by the three regions, commonly holds that clinical trial data of pharmaceuticals should be collected. The three regulatory agencies seek clinical validation of CDx through well-designed clinical studies demonstrating the ability of the test to identify patients likely to respond to the treatment. In other words, findings that the CDx improves patient outcomes when used to guide treatment decisions should be provided. Clinical studies are conducted depending on whether pharmaceuticals and the CDx are co-developed, and bridging studies to evaluate device compatibility can also be conducted. However, even though the MFDS developed guidelines for safety and effectiveness evaluation (2018) after the publication of the guidelines for approval (2015), some sections need to be supplemented. This (2018 guideline) merely lists recommendations for the preparation of clinical trial protocols and can be insufficient as a guide for conducting clinical studies. Therefore, the clinical framework to establish the clinical usefulness of a CDx should be supplemented in future studies.

Demand is increasing in the CDx market, due to the paradigm shift to precision medicine. Accordingly, several countries are establishing continuous regulatory support.[29,32] This can improve patient outcomes and reduce medical costs by ensuring the accuracy and reliability of a CDx and assist with clinical implementation by providing developers with a process for standards required for verification. In response to stricter clinical CDx regulations, manufacturers should put in place measures to promote collaboration between diagnostic companies, pharmaceutical companies, and regulatory agencies for more effective CDx development and approval.[1] However, the enormous data resources needed to establish the framework may take a lot of time, which may delay CDx and drug co-development and approval. CDx verification criteria vary between countries, and implementation of the framework can be difficult in countries or regions with limited infrastructures.[34] However, if an effective internationally harmonized clinical process framework is established for enhanced clinical regulation, it can contribute to providing a more personalized and effective treatment. In conclusion, if studies are conducted to develop an efficient process by establishing a framework for the clinical regulation of CDx based on internationally harmonized regulations, it will facilitate the development and clinical implementation of CDx to enable more effective treatments for patients.

Our study has several strengths. This study reviewed the changing CDx regulations and conducted institutional comparison by country to prepare an efficient plan for clinical tasks. Rather than simply mentioning the need for efficiency measures, considerations for the application of the process were also proposed by analyzing various data. However, this study had a limitation in that it was not possible to analyze the current permit status in Europe. This needs to be done as a future case study if European Database on Medical Devices (EUDAMED) transparency is addressed. In addition, the SWOT analysis was supplemented by accepting the opinions of various stakeholders, including seminars, webinars, and press releases. This study showed that the reinforcement of a framework for complex CDx clinical regulations, which is increasingly important in precision

medicine, can be complemented by establishing an efficient process. Research complementing the complex process can reduce recalls that can raise safety concerns and enable more effective patient treatment and efficient regulatory work by manufacturers by providing a systematic framework.

SUPPLEMENTAL INFORMATION

Supplemental information can be found online at https://doi.org/10.1016/j.omtm.2023.08.008.

ACKNOWLEDGMENTS

This work was supported and funded by the training program for advanced medical device industry professional manpower of Korea Health Industry Development Institute funded by the Ministry of Health and Welfare, Republic of Korea and the Dongguk University Research Fund of 2023 (S-2023-G0001-00044).

AUTHOR CONTRIBUTIONS

Conceptualization, S.L.K. and N.H.K.; investigation, S.L.K., N.H.K. and J.H.W.; data collection, extraction & analysis, S.L.K. and J.H.W.; writing – original draft, review & editing, S.L.K. and J.H.W.; visualization, J.Y.K.; supervision, S.M.K.; project administration, S.M.K.

DECLARATION OF INTERESTS

The authors declare no competing interests.

REFERENCES

1. Keeling, P., Clark, J., and Finucane, S. (2020). Challenges in the clinical implementation of precision medicine companion diagnostics. Expert Rev. Mol. Diagn. *20*, 593–599.
2. Roscoe, D.M., Hu, Y.F., and Philip, R. (2015). Companion diagnostics: a regulatory perspective from the last 5 years of molecular companion diagnostic approvals. Expert Rev. Mol. Diagn. *15*, 869–880.
3. Choi, S.E., and Lim, E.A. (2016). Companion Diagnostics and Targeted Therapy at the Korean Health Technology Assessment Context. J Health Tech Assess *4*, 44–52.
4. Cobbaert, C., Capoluongo, E.D., Vanstapel, F.J., Bossuyt, P.M., Bhattoa, H.P., Nissen, P.H., Orth, M., Streichert, T., Young, I.S., Macintyre, E., et al. (2022). Implementation of the new EU IVD regulation-urgent initiatives are needed to avert impending crisis. Clin. Chem. Lab. Med. *60*, 33–43.
5. U.S. Food & Drug Administration (2019). Class 2 Device Recall Foundationone CDx Test Report. https://www.accessdata.fda.gov/scripts/cdrh/cfdocs/cfRES/res.cfm?id=176246.
6. Jørgensen, J.T. (2016). Companion and complementary diagnostics: Clinical and regulatory perspectives. Trends Cancer *2*, 706–712.
7. Philip, R., Carrington, L., and Chan, M. (2011). US FDA perspective on challenges in co-developing *in vitro* companion diagnostics and targeted cancer therapeutics. Bioanalysis *3*, 383–389.
8. Olsen, D., and Jørgensen, J.T. (2014). Companion diagnostics for targeted cancer drugs - Clinical and regulatory aspects. Front. Oncol. *4*, 1–8.
9. Lee, E.Y., and Shen, H.C.J. (2015). Regulatory considerations for companion diagnostic devices. Biomark. Med. *9*, 67–75.
10. Enzmann, H., Meyer, R., and Broich, K. (2016). The new EU regulation on *in vitro* diagnostics: potential issues at the interface of medicines and companion diagnostics. Biomark. Med. *10*, 1261–1268.
11. Valla, V., Alzabin, S., Koukoura, A., Lewis, A., Nielsen, A.A., and Vassiliadis, E. (2021). Companion Diagnostics: State of the Art and New Regulations. Biomark. Insights *16*. 11772719211047763.
12. APAC Med (2022). Ensuring patient access and supply continuity during EU IVDR transition-A regional trade association perspective. Webinar. KMDIA.
13. U.S. Food & Drug Administration (2023). List of Cleared or Approved Companion Diagnostic Devices (In Vitro and Imaging Tools). https://www.fda.gov/medical-devices/in-vitro-diagnostics/list-cleared-or-approved-companion-diagnostic-devices-in-vitro-and-imaging-tools.
14. Ministry of Food and Drug Safety. Electronic Civil Complaint Desk for Medical Devices. https://udiportal.mfds.go.kr/msismext/emd/min/mainView.do.
15. U.S. Food & Drug Administration (2013). Design Considerations for Pivotal Clinical Investigations for Medical Devices. https://www.fda.gov/regulatory-information/search-fda-guidance-documents/design-considerations-pivotal-clinical-investigations-medical-devices.
16. U.S. Food & Drug Administration (2017). Investigational IVDs Used in Clinical Investigations of Therapeutic Products. https://www.fda.gov/regulatory-information/search-fda-guidance-documents/investigational-ivds-used-clinical-investigations-therapeutic-products.
17. U.S. Food & Drug Administration (2014). In vitro companion diagnostic devices. https://www.fda.gov/regulatory-information/search-fda-guidance-documents/in-vitro-companion-diagnostic-devices.
18. U.S. Food & Drug Administration (2016). Principles for Codevelopment of an in Vitro Companion Diagnostic Device with a Therapeutic Product. https://www.fda.gov/regulatory-information/search-fda-guidance-documents/principles-codevelopment-in-vitro-companion-diagnostic-device-therapeutic-product.
19. European Medicines Agency (2017). Regulation (EU) 2017/746 of the European parliament and of the council of 5 April 2017 on *in vitro* diagnostic medical devices and repealing directive 98/79/EC and commission decision 2010/227/EU. https://eur-lex.europa.eu/legal-content/EN/TXT/?uri=CELEX%3A32017R0746&qid=1682560553487.
20. European Medicines Agency (2022). Guidance on the Procedural Aspects for the Consultation to the European Medicines Agency by a Notified Body on Companion Diagnostics. https://www.ema.europa.eu/en/documents/scientific-guideline/guidance-procedural-aspects-consultation-european-medicines-agency-notified-body-companion_en.pdf.
21. European Medicines Agency (2009). Guideline on Clinical Evaluation of Diagnostic Agents. https://www.ema.europa.eu/en/clinical-evaluation-diagnostic-agents-scientific-guideline.
22. Ministry of Food and Drug Safety (2020). Guidelines of Clinical Performance Test for in Vitro Diagnostic Medical Devices. https://www.mfds.go.kr/brd/m_1060/view.do?seq=14683&srchFr=&srchTo=&srchWord=&srchTp=0&itm_seq_1=0&itm_seq_2=0&multi_itm_seq=0&company_cd=&company_nm=&Data_stts_gubun=C1004&page=1.
23. Ministry of Food and Drug Safety (2018). Guidelines of Safety, Performance and Clinical Trial Evaluation for Companion Diagnostic Medical Devices Using Liquid Biopsy. https://www.khidi.or.kr/board/view?pageNum=30&rowCnt=20&no1=1478&linkId=48711129&refMenuId=MENU01525&menuId=MENU01521&maxIndex=00487339719998&minIndex=00460907489998&schType=0&schText=&schStartDate=&schEndDate=&boardStyle=&categoryId=&continent=&country=/.
24. Ministry of Food and Drug Safety (2015). Guidelines of Approval and Review for in Vitro Companion Diagnostic Devices. https://www.nifds.go.kr/brd/m_15/view.do?seq=13131/.
25. Donawa, M. (2014). What You Need to Know about FDA IVD Test Requirements (European Medical Device Technology). https://www.donawa.com/wp-content/uploads/2019/06/IVD-article-on-FDA-test-requirementsEMDT-reduced.pdf/.
26. U.S. Food & Drug Administration. 21 CFR 860.7 Determination of Safety and Effectiveness. https://www.accessdata.fda.gov/scripts/cdrh/cfdocs/cfcfr/CFRSearch.cfm?FR=860.7/.
27. Wise, J., Furness, M., McWilliams, S., and Patton, S. (2019). CDx, NGS and regulation: five perspectives from the Pistoia Alliance. Drug Discov. Today *24*, 2120–2125.
28. M.edT.ech Europe (2021). Clinical Evidence Requirements for CE Certification under the in Vitro Diagnostic Regulation in the European Union. https://www.

medtecheurope.org/resource-library/clinical-evidence-requirements-for-ce-certification-under-the-in-vitro-diagnostic-regulation-in-the-european-union/.

29. Han, S.H. (2022). Pay Attention to the Mid- to Long-Term Growth Potential of the in Vitro Diagnostics Industry. http://money2.daishin.com/pdf/out/intranet_data/Product/ResearchCenter/Report/2022/05/44184_Diagnostics_Industries_PM-v2.pdf/.

30. Choi, H.J. (2021). Companion diagnostics. Innovative growth item report. https://bit.ly/3BllGh6/.

31. Kim, J.W., and Kang, M.R. (2022). Global *in vitro* diagnostic(IVD) trends. Bio Econ. Brief *157*, 1–7. https://www.koreabio.org/board/board.php?bo_table=brief/.

32. Bush, T. (2019). Global companion diagnostic (CDx) registration: Clinical evidence and regulatory pathways. In 24th AHWP Annual Meeting, http://www.ahwp.info/sites/default/files/20191111%20-%2003%20-%20Track202%20-%20Regulatory%20pathways%20and%20clinical%20evidence%20requirements%20for%20Companion%20Diagnostics.pdf/.

33. Heinzelmann, E. (2018). The new, stringent MDR and IVDR regulations: Viewing this change as an opportunity. Chimia *72*, 430–431.

34. FEAM EUROPEAN BIOMEDICAL POLICY FORUM (2018). Companion Diagnostics and Precision Medicine: Regulatory and Uptake Barriers to Patient Access. https://www.feam.eu/wp-content/uploads/FEAM-Forum_Round-table-PM_Report_Final.pdf/.

35. National Institute of Medical Device Safety Information (2022). Magazine of Innovative Medical Device R&D Information, *5*. https://www.nids.or.kr/board/view.jsp?page=IMRS&flag=view&data_seq=10483&i_current_page=1&mnu=1&tabno=0&category=1/.

36. European Federation of Pharmaceutical Industries and Associations (2020). Determining the Path for Assessment of a Companion Diagnostic (CDx) under the in Vitro Diagnostic Medical Devices Regulation. https://www.efpia.eu/media/554434/2020_05_27_efpia-mte_determining-the-path-for-assessment-of-cdx-under-ivdr_final.pdf/.

37. U.S. Food & Drug Administration (2013). Paving the Way for Personalized Medicine. https://www.fdanews.com/ext/resources/files/10/10-28-13-Personalized-Medicine.pdf/.

38. Cowan, E.P.. Performance Studies. https://www.fda.gov/files/vaccines%2C%20blood%20%26%20biologics/published/Day-2b_Review-of-Information-Submitted-2.pdf/.

39. Makrocare LLC. (2020). IVD Clinical Performance Studies for FDA & EU. https://www.makrocare.com/blog/ivd-clinical-performance-studies-for-fda-eu/.

40. BSI (2020). Introduction to the IVDR Revision (Web Seminar). https://www.youtube.com/watch?v=3VwX0BXNxN8.

OMTM, Volume *30*

Supplemental information

Necessity of strengthening the current clinical regulatory for companion diagnostics: An institutional comparison of the FDA, EMA, and MFDS

Su Lim Kang, Jae Hyun Woo, Na Hyeon Kim, Ji Yean Kwon, and Sung Min Kim

Supplemental Material

Table S1. Approved CDx list in FDA

Table S2. Approved CDx list in MFDS

Table S3. Factors that may affect clinical investigation design

	[21 CFR 860.7] Determination of safety and effectiveness
**Purpose**	- A clear statement of the purpose of the study
**population selection**	- Confirmation of the suitability of the subject for the purpose of the study, criteria for the condition to be treated or diagnosed, laboratory tests, if applicable, and, in the case of a device intended to prevent a disease or condition, evidence of susceptibility to and exposure to that condition<br>- Allocate subjects in a way that minimizes possible bias<br>- Demonstration of comparability between the test group and all control groups of relevant variables such as gender, severity or duration of disease, and non-instrumental method
**Parameter**	- Description of methods for observing and recording outcomes, including variables measured, quantification, assessment of subjects' responses, and procedures chosen to minimize subject and observer bias
**Blinding**	- Comparison of treatment or diagnostic results with a control group in a manner that allows for quantitative evaluation<br>- A description of the methods specified and used to minimize the bias of observers and data analysts<br>- Document the level and method of blinding<br>: No treatment. Comparison of objective outcomes in comparable treatment and noncomparative patient groups, provided that objective measures of efficacy are available and the placebo effect is negligible<br>: Placebo control. Where there may be a placebo effect in device use, comparison with an ineffective device used under designed conditions that are as close as possible to the conditions of use under investigation<br>: Active treatment control. Comparison when an effective regimen can be used for comparison<br>: Historical control. Quantitative comparison with past prior experience in the case of specific circumstances associated with high mortality and predictable disease or duration, signs and symptoms of severity, and predictable prophylaxis
**Analysis of results**	- Summary of methods of analysis and evaluation of data derived from the study, including appropriate statistical methods used

Table S4. Diagnostic clinical performance study

	Diagnostic clinical performance study
**Intended Use**	- The study design of the diagnostic clinical performance study depends on the intended use of the device.<br>- Therefore, it should be evaluated in the following items corresponding to the purpose of use.<br>• What the device measures or detects<br>• What the device reports<br>• The cell, tissue, organ, part or system examined<br>• Sample Source, Sample Type, and Sample Matrix (-ces)<br>• How to use the device (following the directions for use)<br>• When using the device (conditions of use)<br>• the person using the device (operator or target user)<br>• what for (goal condition)<br>• Who (target population) the device is being used for
**Reference Standard**	- Clinical reference standards for regulatory purposes are the best method available to establish the true condition of subjects<br>- Clinical reference standards should be established before starting the study, and appropriate standards should be used and interpreted as they are updated with technological advances
**Subject selection and sample collection**	- Subject preparation, specimen collection, storage, and handling procedures are important components that must be fully described in the study protocol<br>- In a prospective study design, a pre-specified protocol is used. These protocols pre-specify the study design, including inclusion/exclusion criteria, subject recruitment and selection methods, testing protocols, and analysis methods<br>- In a retrospective study design, the researcher searches for subjects through available data, specimens, images, or other information stored on media or devices<br>- This includes going to tertiary care centers to obtain samples or using enrollment data from previous studies with long-term follow-up
**Blinding**	- Results from clinical reference standards or other diagnostic assessments should not be recognized and should be blinded, as multiple assessments and users/readers may be involved
**Researcher's proficiency**	- Skills through work training, research knowledge, and experience in reading results may affect the performance of the device.<br>- Therefore, the clinical research protocol should consider the performance variability according to the skill level of the researcher handling the device, and additional performance research by other researchers may be required.<br>- Sponsors may be required to document researcher training.

Table S5. Intended use and performance indicator of CDx

Intended use	Performance indicator	Subject of study	Type of study
**Treatment stratification**	Patient outcome measurements and interaction analysis	all new entrants (All patients receiving drug therapy)	Prospective randomized and retrospective trials, correlational trials
**Treatment selection**		Biomarker positive patients	

Table S6. Performance evaluation according to the IVDR

	IVDR Annex 13. Part A 1. Performance evaluation
**Plan**	- Specifications for the intended purpose of the device (specification)<br>- Equipment characteristics (Annex 1. Chapter 2, Section 9, Chapter 3, Section 20.4.1 (c))<br>- Specifications of the analyte or marker determined by the device<br>- Specifications for the purpose of the device (specification)<br>- Identification of certified reference materials or standard measurement procedures to enable quantitative traceability<br>- Clear identification of specific target patient populations with clear symptoms, limitations and contraindications to use (specific drugs or treatments)<br>- Annex 1. Identification of general safety and performance requirements specified in Sections 1 to 9 (based on relevant scientific justification and analytical/clinical performance data)<br>- Description of methods used to assess analytical/clinical performance, limitations of the device, and description of accompanying information.<br>- Description of state-of-the-art technology (including relevant standards, CS, guidelines or best regulatory documents)<br>- Indication and description of the parameters used to determine the acceptability of the risk-benefit ratio for the intended use of the device and for its analytical/clinical performance, based on the latest knowledge in the field of medicine<br>- Identification and description of reference databases and other data sources used as a basis for decision-making, for software qualifying as a device<br>- Outline of the different stages of development, including indication of key timelines and description of possible acceptance criteria (including sequence and means of determining scientific validity, analytical/clinical performance)<br>- PMPF plan specified in Annex Part B
**Scientific validity**	- Relevant information on the scientific justification of devices measuring the same analyte or marker<br>- Scientific (peer reviewed) literature<br>- Relevant professional associations, unanimously agreed expert opinions/positions<br>- Results of proof-of-concept studies<br>- Results of clinical performance studies
**Analytical performance**	- As a general rule, always validated on the basis of analytical performance studies<br>- In the absence of a comparative method, other approaches may be used if proven appropriate<br>- In the absence of an approach, clinical performance studies comparing the performance of new devices with the latest clinical regulations are required
**Clinical performance**	- Proven based on one or more of clinical performance studies, scientific (peer reviewed) literature, and published experience from routine diagnostic testing<br>- Unless justification for reliance on other sources of clinical performance data is provided<br>- Proven and documented in clinical performance reports

Table S7. Clinical performance study according to the IVDR

IVDR Annex 13. Part A 2. Clinical performance studies	
**Purpose**	- Establish or verify performance of a device that cannot be judged by experience gained through analytical performance studies, literature, and routine diagnostic testing<br>- Used to demonstrate compliance with general safety and performance requirements related to clinical performance<br>- Data obtained from conducting clinical performance studies should be used in the performance evaluation process and should be part of the clinical evidence of the device
**Ethical considerations**	- Each phase of the clinical performance study is conducted in accordance with recognized ethical principles
**Design type**	- Designed in such a way as to maximize the relevance of data while minimizing bias
**Plan**	- Should be conducted based on the Clinical Performance Study Plan (CPSP) and should define the rationale, objectives, design and proposed analysis, methods, monitoring, conduct and record keeping<br>- Include the following information<br>: Single identification number<br>: Identification number of the client (name of the client, address of the registered office, contact information, etc.)<br>: Information about the researcher (contact information, research institute information)<br>: Study start date and planned duration<br>: Device identification information and description, use, analyte or label, quantitative traceability, manufacturer<br>: Information on the type of specimen being studied<br>: Overall summary of the study, design type, research objective, hypotheses, diagnosis and reference to state-of-the-art technology in medicine<br>: Expected risks and benefits of the device, description of the study, exceptions to studies using residual specimens, related medical procedures and patient management<br>: Instructions for use of the device, test protocols, training and experience required by the user, appropriate calibration procedures and controls, indications of other devices, medical devices, pharmaceuticals, or other items to be included or excluded, and to other comparable devices or methods used as references. description of<br>: Description of study design and validity, scientific robustness, detailed description of measures to minimize bias, and management of potential confounding factors<br>: Analytical performance, justification for omissions<br>: Clinical performance parameters, justification for any omissions, exceptions to studies using residual samples, specified clinical outcome/endpoints (primary/secondary) and their validity, potential impact on personal health and public health management decisions<br>: Population information (subjects, selection criteria, population size, vulnerable subjects, etc.)<br>: Information on the use of data from residual samples, gene or tissue banks, patient or disease registries, etc., description of reliability, representativeness and statistical analysis approaches, assurance of methods for determining the clinical veracity of patient samples.<br>: Monitoring plan

	: Data Management<br>: Decision algorithm<br>: Policy on CPSP Modifications, Deviations from CPSP, and Clear Prohibition of Waiver<br>: Responsibility for explaining the device (management of access to the device, follow-up, recovery of the device)<br>: Statement of Commitment to Compliance with Ethical Principles, Good Clinical Practice Principles, and Applicable Regulatory Requirements<br>: Description of the informed consent process (consent form)<br>: Safety Recording and Reporting Procedures<br>: Criteria and Procedures for Suspension or Early Termination<br>: Criteria and procedures for follow-up of subjects after completion, discontinuation or early termination, follow-up procedures for subjects who withdrew informed consent, and procedures for subjects who did not receive follow-up<br>: Procedure for delivering test results to the outside<br>: Policies related to reporting and presentation of results in accordance with legal requirements and ethical principles<br>: List of technical/functional features of the device<br>: References
**Report**	- Include documented information about the protocol plan, results and conclusions, along with negative findings, in a report signed by a physician or other authorized person<br>- Transparent, unbiased, clinically relevant results and conclusions<br>- Include enough information that an independent party can understand it without consulting other documents<br>- Include changes or non-compliance with protocols and data exclusions where appropriate

Table S8. Analytical and clinical performance evaluation indicators

Analytical performance	Clinical performance
Accuracy<br>Precision<br>Specificity<br>Measuring range and linear interval, such as quantitation range or detection limit<br>Analytical cutoff<br>Standard material<br>Information on the sample being collected<br>Possibility of occurrence of non-specific reactions and methods of suppression<br>Possibility of contamination and methods of elimination	Positive or negative consistency between the applied product and the licensed product or existing test method<br>Drug responsiveness using IVD-CDx

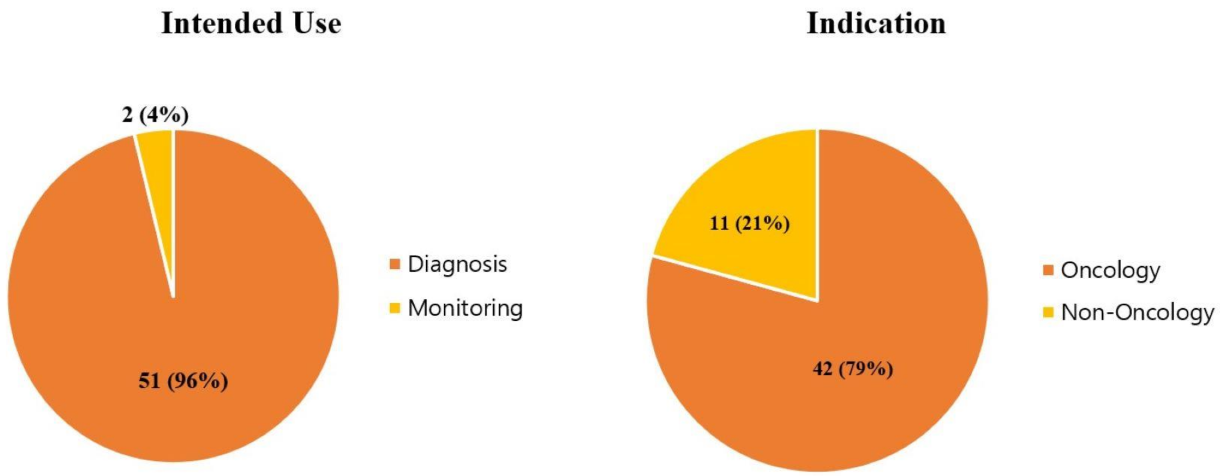

Figure S1. Approval status in FDA according to intended use and indication type. (a): Intended use. (b): Indication type. These graphs are analysis of CDx products approved by the FDA based on intended use and indication type. As a result of analyzing the intended use according to the definition of the device, 96% (51/53) were used to identify patients through disease diagnosis and 4% (2/53) were used to monitor a disease. Depending on the type of indication, 79% (42/53) appeared as a device for diagnosing cancer or tumor.

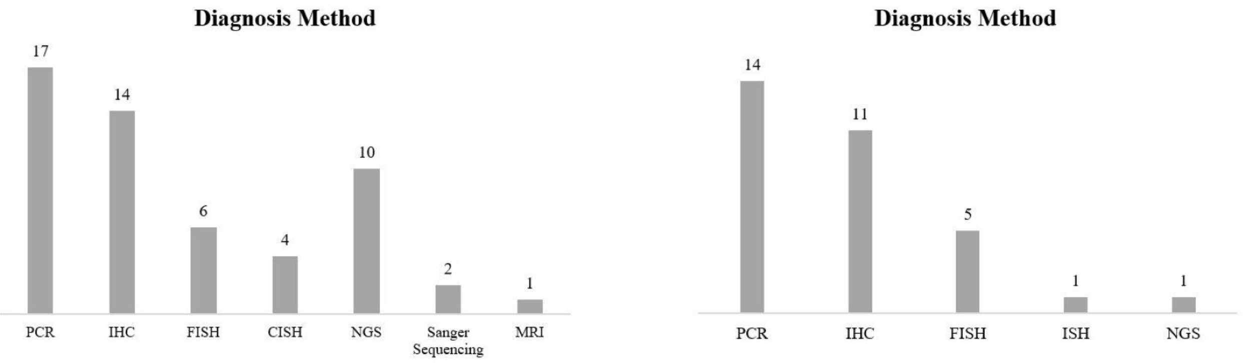

Figure S2. Approval status according to the diagnosis method. (a): FDA. (b): MFDS. These graphs are analysis of CDx products approved by the FDA and MFDS based on diagnosis method. Polymerase chain reaction (PCR) technology (32%) was applied the most frequently as the diagnosis method in FDA. All products in MFDS were used to identify patients by diagnosing cancer of oncology. In Particular, PCR technology (44%) was the most frequently applied diagnosis method.
